# Neuroinflammation, Bone Marrow Stem Cells, and Chronic Pain

**DOI:** 10.3389/fimmu.2017.01014

**Published:** 2017-08-21

**Authors:** Yul Huh, Ru-Rong Ji, Gang Chen

**Affiliations:** ^1^Department of Anesthesiology, Duke University Medical Center, Durham, NC, United States; ^2^Department of Neurobiology, Duke University Medical Center, Durham, NC, United States; ^3^Key Laboratory of Neuroregeneration of Jiangsu and Ministry of Education, Co-Innovation Center of Neuroregeneration, Nantong University, Nantong, Jiangsu, China

**Keywords:** neuroinflammation, bone marrow stem cells, chronic pain, treatment, transforming growth factor beta

## Abstract

Current treatments for chronic pain, such as inflammatory pain, neuropathic pain, and cancer pain are insufficient and cause severe side effects. Mounting evidence suggests that neuroinflammation in the peripheral and central nervous system (PNS and CNS) plays a pivotal role in the genesis and maintenance of chronic pain. Characteristic features of neuroinflammation in chronic pain conditions include infiltration of immune cells into the PNS [e.g., the sciatic nerve and dorsal root ganglion (DRG)], activation of glial cells such as microglia and astrocytes in the CNS (spinal cord and brain), and production and secretion of pro-inflammatory cytokines and chemokines [TNF, interleukin (IL)-1β, IL-6, CCL2, and CXCL1]. Recent studies suggest that bone marrow stem cells or bone marrow stromal cells (BMSCs) produce powerful analgesic effects in animal models of inflammatory pain, neuropathic pain, and cancer pain. We recently demonstrated that intrathecal injection of BMSCs resulted in a long-term relief of neuropathic pain for several weeks after peripheral nerve injury. Strikingly, this analgesic effect is mediated by the anti-inflammatory cytokine transforming growth factor beta secreted from BMSCs. Additionally, BMSCs exhibit potent modulation of neuroinflammation, by inhibiting monocyte infiltration, glial activation, and cytokine/chemokine production in the DRG and spinal cord. Thus, BMSCs control chronic pain by regulation of neuroinflammation in the PNS and CNS *via* paracrine signaling. In this review, we discuss the similar results from different laboratories of remarkable anti-nociceptive efficacy of BMSCs in animal and clinical studies. We also discuss the mechanisms by which BMSCs control neuroinflammation and chronic pain and how these cells specifically migrate to damaged tissues.

## Introduction

Whereas acute pain can bring attention to the body of possible injuries and is normally a protective sensation, chronic pain does not convey any useful information and has no biological benefits. It only gives people a feeling of discomfort but does not play an active role in wound healing. Chronic pain can persist for months to years, even after the primary injury or inflammation has healed. Chronic pain is a major clinical problem that affects up to 30% of adults in the world (1, 2). Chronic pain costs the US economy more than $600 billion per year in healthcare expenditures, disability payments, and lost productivity (from American Pain Society) (2). Medications, massage therapy, acupuncture, electrical stimulation, nerve blocks, and surgery are some traditional therapies for chronic pain. These methods can be powerful and effective for some patients. However, there are no drugs or treatments currently available that treat chronic pain in a complete and definitive way. Growing evidence suggests that bidirectional signaling between the immune system and the nervous system contributes to the development and maintenance of chronic pain (3, 4). Neuroinflammation results from the activation of glial cells in the peripheral nervous system including Schwann cells and satellite glial cells, in the central nervous system including microglia, astrocytes, and oligodendrocytes, as well as the activation of immune cells including resident mast cells and macrophages and infiltrating neutrophils and T cells.

The production of glial and pro-inflammatory mediators (e.g., cytokines, chemokines, trophic factors, neurotransmitters, and lipid mediators) modulates pain sensitivity, with persistent glial and immune cell activation and interaction with neurons leading to the development of peripheral and central sensitization, and induction of chronic pain conditions. In the periphery, an inflammatory milieu of interleukin (IL)-1β, TNFα, bradykinin, SP, CGRP, NGF, and prostaglandins is released by resident and infiltrating immune cells as well as from sensory nerve terminals (5). In the spinal cord, bidirectional signaling between neurons and glia regulated by chemokines (CXCL1, CCL2, and CX3CL1), proteases [metalloproteinase (MMP)-9, cathepsin S, and caspase 6], and the WNT signaling pathway involve in neuroinflammation and chronic pain sensitization (3). Thus, neuroinflammation is associated with various painful insults and pathologies which include neuropathic pain from nerve and spinal cord injury, inflammatory pain caused by arthritis, cancer pain, and pain caused by drug therapy.

Targeting the specific processes and molecules involved in neuroinflammation provides new therapeutic opportunities for chronic pain. For instance, inhibiting microglial response with minocycline and p38 MAP kinase inhibitor has been proven to prevent the initiation of neuropathic pain in rodents (6–8). Targeting activated astrocyte signaling through connexin-43 and CXCL1 inhibition has also demonstrated the ability to reverse established chronic neuropathic pain following peripheral nerve injury (5, 9). Pro-resolution lipid mediators (PRLMs) including resolvins, protectins, and lipoxins are new targets for treating chronic pain as they possess potent anti-inflammatory and anti-nociceptive properties through action on neurons, immune cells, and glial cells, but importantly do not suppress immune function. By protecting the body from bacterial and viral infection, PRLMs are poised as one of the most effective treatments for preventing surgical or trauma-induced chronic pain (3, 10).

## BMSCs Produce Pain Relief in Animal Models

Recent studies have revealed the therapeutic potential of bone marrow stromal cells/bone mesenchymal stem cells (BMSCs) for chronic pain (11–14). BMSCs are a heterogeneous population of stromal cells present in bone marrow that give rise to various tissues throughout the body (15). Due to their strong immunosuppressive properties, BMSCs can be used in both autologous and heterologous transplantation without the need for immunesuppressive agents (16). BMSCs were originally considered by researchers as stem cells to reconstruct damaged and/or diseased tissues (15). However, recent studies have shown that BMSCs can affect a variety of physiological and pathophysiological processes, including immune and inflammatory responses, by releasing cytokines, chemokines, and trophic factors (17). Here, we review the immunomodulatory effects of BMSCs and the possible mechanisms of action. We also consider the implications of these data for clinical studies of BMSCs in the management of chronic pain.

The ability of BMSCs to alter the inflammatory milieu has made BMSCs an attractive treatment possibility for various painful states such as inflammatory pain, neuropathic pain, and cancer pain. Fortunately, many studies have reported pain relief in animal pain models with BMSC treatment (Table 1).

**Table 1:** Pain relief by BMSCs under different injury and injection conditions.

Year	Reference	Disease (model) and species	Cell source	Number of cells	Delivery site	Effects on pain
2007	Musolino et al. (41)	SLNC, rat	Rat	2 × 10^5^	Intraganglionic	Prevention of mechanical and thermal allodynia
2007	Klass et al. (50)	CCI, rat	Rat	1 × 10^7^	Intravenous	Improvement of mechanical allodynia and thermal hyperalgesia
2008	Shibata et al. (45)	STZ-induced diabetes, rat	Rat	1 × 10^6^	Injection in the hind limb skeletal muscle	Improvement of hypoalgesia
2009	Abrams et al. (42)	Spinal cord injury, rat	Rat	3 × 10^5^	Injury site	Improvement of mechanical allodynia, no effect on thermal hyperalgesia
2010	Siniscalco et al. (44)	SNI, mouse	Human	5 × 10^4^	Lateral cerebral ventricle	Improvement of mechanical allodynia and thermal hyperalgesia
2011	Siniscalco et al. (51)	SNI, mouse	Human	2 × 10^6^	Intravenous	Improvement of mechanical allodynia and thermal hyperalgesia
2011	Orozco et al. (34)	Degenerative disk disease, human	Human	10 ± 5 × 10^6^ per disk	Intradisc injection	Decrease in pain
2011	Guo et al. (11)	Chronic orofacial pain, rat	Rat	1.5 × 10^3–6^; 1.5~3.75 × 10^5^	Intravenous; injury site	Reversed mechanical hypersensitivity
2011	Naruse et al. (46)	STZ-induced diabetes, rat	Rat	1 × 10^6^	Injection in the hind limb skeletal muscle	Improves mechanical hyperalgesia, cold allodynia
2014	van Buul et al. (12)	Osteoarthritis, rat	Rat	1 × 10^6^ per joint	Intra-articular injection	Decrease in pain
2014	Zhang, et al. (47)	SNL, rat	Rat	1 × 10^5^	Intrathecal injection	Improvement of mechanical allodynia
2015	Chen et al. (14)	CCI, SNI, mouse	Mouse	1~2.5 × 10^5^	Intrathecal injection	Suppress neuropathic pain
2016	Pettine et al. (33)	Degenerative disk disease, human	Human	2–4 × 10^8^ nucleated cells per disk	Intradisc injection	Decrease in pain
2016	Yousefifard et al. (43)	Spinal cord injury, rat	Human	1 × 10^6^	Injury site	Improvement of mechanical and cold allodynia; mechanical and thermal hyperalgesia
2016	Guo et al (52)	TL, SNL, CCI-ION, rat, and mice	Rat, human	1.5 × 10^6^	Intravenous; injury site	Improvement of mechanical and thermal hyperalgesia; suppress aversive behavior
2017	Li et al. (48)	SNL, rat	Rat	2.5 × 10^6^	Intrathecal injection	Improvement of mechanical allodynia and thermal hyperalgesia
2017	Fischer et al. (49)	TNI, rat	Rat	2.5 × 10^5^	Intrathecal injection	Improvement of mechanical hyperalgesia

*CCI, chronic constriction injury; SLNC, single ligature nerve constriction; SNI, spared nerve injury; SNL, spinal nerve ligation; STZ, streptozotocin; TL, tendon ligation; CCI-ION, chronic constriction injury of the infraorbital nerve; TNI, tibial nerve injury*.

### Inflammatory Pain

Osteoarthritis (OA) is a form of inflammatory pain with a significant impact on quality of life. At present only a few therapies are effective for OA patients, with most of them designed to relieve pain, control inflammation, and improve function (18). BMSC-based therapeutic efforts to treat OA have been well documented in different animal models including murine (19), rabbit (20, 21), sheep (22, 23), and horse (24). Autologous or allogeneic autologous BMSCs were injected into the joints of tested animals. Transplanted BMSCs were shown to reduce the progression of OA by controlling the inflammation, reducing cartilage loss, and improving cartilage content (25–27). Initially, BMSCs were considered the ideal source of direct regeneration of the articular surface. Recently, an increasing number of studies have shown that the major benefits of BMSCs come from paracrine activity (12, 25, 26, 28). Injections of MSCs have led to documented joint tissue regeneration; however, some studies have found that native cells are what primarily comprise reconstituted tissues with few transplanted cells contributing to regenerated tissue (23). Other studies have shown that the cell signaling milieu changes following delivery of MSCs specifically with a subsequent increase in host type II collagen production (19). Together, these factors suggest that MSCs may be coordinating a state of repair rather than directly replacing damaged tissues. This falls in line with the anti-inflammatory and immunomodulatory roles of MSCs.

Intervertebral disk degeneration is directly related to chronic inflammatory back pain. Cytokines in the degenerative tissue cause pain directly by enhancing protease activity (29). At present, there is no medication or treatment that can treat chronic back pain in a complete way without the risk of major side effects. Non-invasive therapies offer limited efficacy in the treatment of pain, and although surgical removal of the disk can relieve pain immediately, degeneration of adjacent segments can occur with the subsequent return of pain. In order to avoid recurrent pain and invasive surgical procedures, BMSC-based therapy is now being studied as a promising approach for the repair of degenerative intervertebral disks (30–32). In a similar role to their use in treating OA, BMSCs can also modulate the inflammatory microenvironment from intervertebral disk injury, and reduce inflammatory pain *via* paracrine pathways (31, 33, 34).

Inflammatory bowel disease (IBD) is marked by recurring and idiopathic intestinal inflammation, which can lead to significant morbidity and potential mortality. Symptoms include abdominal pain, cramps, bloody stool, and persistent diarrhea or constipation, all of which significantly impair a patient’s quality of life (35). However, due to a lack of understandings of the origins of IBD, current treatment strategies fail to treat the root causes of IBD and mainly combat the symptoms of the disease. The treatments are further limited by a lack of efficacy as well as toxic and adverse side effect profiles (36). Recently, BMSCs have attracted increasing amounts of attention for the treatment of IBD (37–39). Intravenous injection of BMSCs in rats reduced the damage of the intestinal mucosal barrier, led to the down-regulation of zona occludens 1 expression, and reduced the intestinal damage mediated by a TNFα-mediated mechanism (40).

### Neuropathic Pain

Neuropathic pain is a rather stubborn pain induced by nerve injury and can last for months to years, even after the primary tissue damage has healed. Current treatments for neuropathic pain are insufficient. However, in the context of neuropathic pain, transplantation of BMSCs has been shown to reduce the progress of neuropathic pain. In a variety of neuropathic pain models, BMSCs were injected directly into the lesion site [*via* intraganglionic (41), intraspinal (13, 42, 43), intrabrain (44), intramuscular (11, 45, 46), intrathecal injection (14, 47–49), or sytemic intravenous administration (11, 50–52)]. For example, intravenous injection of BMSCs reduced mechanical allodynia and thermal hyperalgesia in rodent chronic constriction injury (CCI) models of the sciatic (50) and infraorbital nerve (11, 52), as well as in spared nerve injury (SNI) models (51). Intramuscular injection of BMSCs reduced mechanical allodynia and cold pain in an STZ-induced diabetic model (45, 46) and also in an infraorbital nerve CCI model of orofacial pain (11). Intraspinal injection of BMSCs reduced mechanical allodynia and thermal hyperalgesia in spinal cord injury models of rats (42) and mice (13). Intraganglionic injections of BMSCs improved mechanical allodynia and thermal hyperalgesia in a rat single ligature nerve constriction model (41). Intrathecal injections of BMSCs improved mechanical allodynia and thermal hyperalgesia in a rat SNL model (47, 48).

In our recent paper (14), we demonstrated long-term analgesic relief of neuropathic pain in mice after a single intrathecal injection of BMSCs. Intrathecal administration of 250,000 BMSCs alleviated symptoms of early- and late-phase neuropathic pain including allodynia and hyperalgesia, for several weeks in mice nerve injury models, including CCI and SNI. In addition, intrathecally administered BMSCs also alleviated CCI-induced ongoing pain. Furthermore, intrathecal BMSCs protected dorsal root ganglion (DRG) neurons from axonal injury and inhibited neuroinflammation in both DRGs and spinal cord tissues in CCI mice. Intrathecal BMSCs specifically target injured dorsal root ganglia *via* a CXCL12/CXCR4 interaction. Nerve injury-induced CXCL12 up-regulation in the injured L4–L5 DRGs leads to the trafficking of CXCR4-expressing BMSCs specifically to the injured DRGs. Intrathecal BMSCs inhibit neuropathic pain *via* transforming growth factor beta (TGF-β)1 secretion, which can be detected in the CSF. The analgesic effect of BMSCs was also reversed by TGF-β1 antibody but not by IL-10 antibody neutralization. Additionally, intrathecal injection of exogenous TGF-β1, at very low doses (1–10 ng) elicited potent analgesic effects in a neuropathic pain model. BMSCs can survive in DRGs for several months, without converting to other cell types, after which they disappeared from DRGs.

### Cancer Pain

Cancer development is often associated with chronic inflammation (53). Although BMSCs have a potential anti-inflammatory effect, the exact role of BMSCs in tumor development remains controversial in the literature (54). Many studies have shown that BMSCs exhibit anti-tumor effects and inhibit tumor growth (55, 56), whereas other studies have suggested the role of pro-tumor effects (57, 58). In general, early-stage cancers often do not cause pain unless there are metastases to bone (59). To date, there is little literature showing the use of BMSCs to treat pain caused by the tumor itself. However, BMSCs have been found to treat chronic visceral pain induced by radiotherapy (60). Radiotherapy is a common type of cancer treatment. However, clinical studies have shown that about 50% of patients who receive radiotherapy have chronic visceral pain or tenesmus. Because of a cross-sensitization between visceral organs, cross sensitivity may amplify incoming signals and cause exacerbation of pain. Intravenously administered BMSCs 4 weeks following radiation treatments induced a time-dependent reversion of the visceral allodynia with a decrease in the anatomical interactions between mast cells and PGP9.5+ nerve fibers. Additionally, MSC treatment has the ability to limit colonic ulceration induced by radiation, a benefit that is not conferred by ketotifen (60).

## Route of BMSCs Administration

Intrathecal administration is safe and there are several FDA-approved drugs for intrathecal injection. Several clinical trials show that intrathecal injection of BMSCs does not cause adverse health issues up to 12 months after treatment. Given the protection of the spinal/brain blood barrier, intrathecal administration of BMSCs can evade host immune responses. Thus, the intrathecal route can target a common pain pathway, produce long-term survival of BMSCs, and in turn provide long-term pain relief. In contrast, systemic administration such as through intravenous injection requires large numbers of BMSCs as most of the injected BMSCs will gather in the pulmonary capillaries and survive for a few days following injection (61–63). Furthermore, local BMSC injections, such as joint injections or intramuscular injections, can only target a single pain site and with short-term survival of BMSCs. Intraspinal or intraganglionic injections are very invasive procedures and may cause additional trauma which can further compromise injured tissue.

Several studies have shown how short-lived paracrine mechanisms are prominent amidst the various therapeutic actions of MSCs. Toma et al. (64) conducted a study where human MSCs (hMSCs) tagged with β-galactosidase were injected into the left ventricles of immunodeficient mice. Four days following injection, most of the injected hMSCs were found in the lung, spleen, and liver. Additionally only 0.44% of the injected hMSCs survived, but when compared with surrounding cardiomyocytes, these cells were morphologically indistinguishable. The findings from this study were reflected by other groups which have reported that less than 1% of systemically administered MSC cells survive for more than 1 week and that the secreted factors and not so much the cells themselves are responsible for the benefits of MSC therapy (62, 65).

## Mechanisms and Mediators by Which BMSCs Reduce Pain and Neuroinflammation

**Figure 1:**
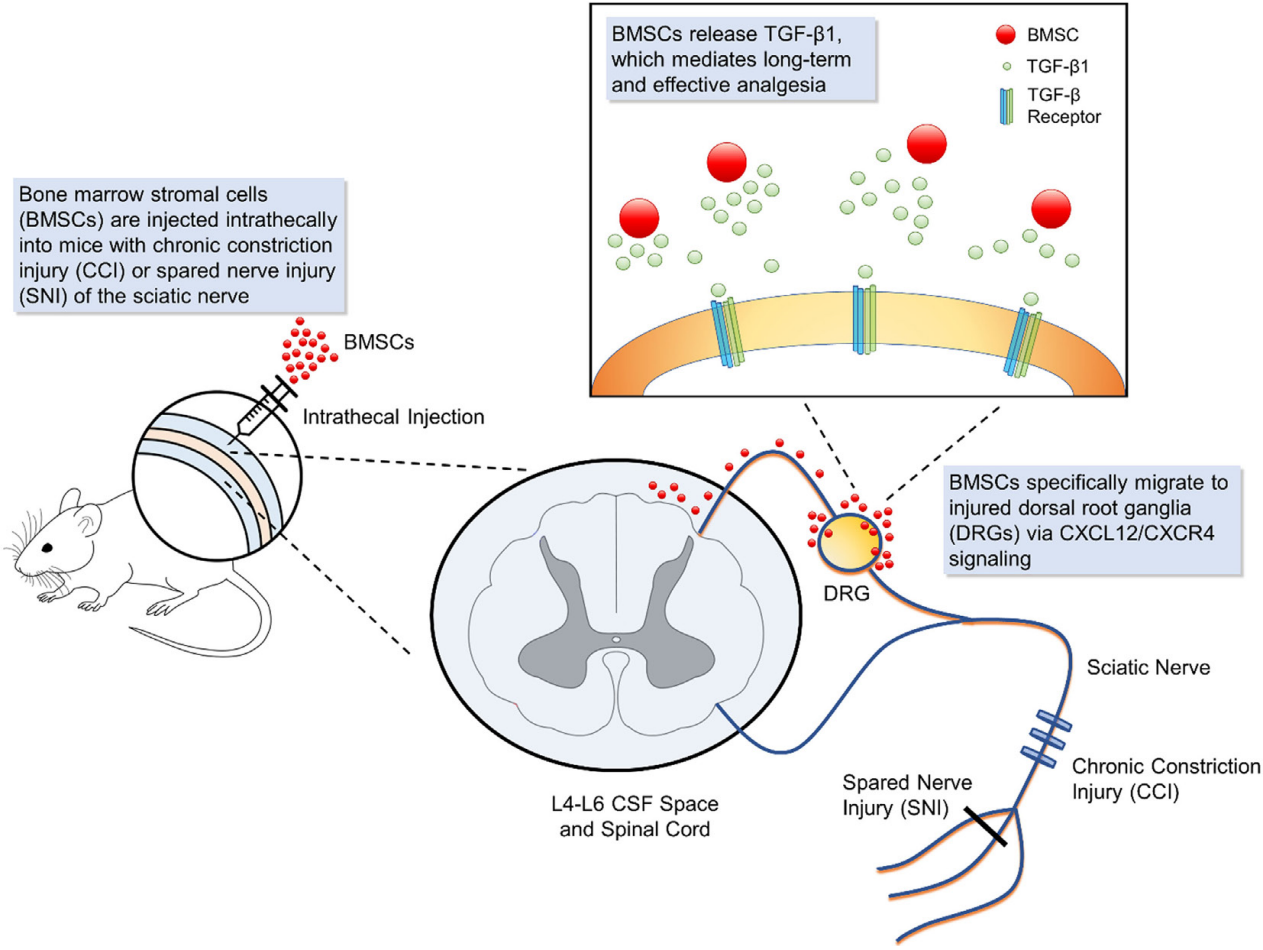
Schematic of bone marrow stromal cell (BMSC) intrathecal injection for treating neuropathic pain. Intrathecal injection introduces bone marrow stromal cells into the cerebrospinal fluid. In mouse models of neuropathic injury of the sciatic nerve, including chronic constriction injury and spared nerve injury, BMSCs expressing CXCR4 specifically migrate to the L4-L6 dorsal root ganglia (DRG) where injured neurons up-regulate the corresponding ligand CXCL12. At the DRG, BMSCs secrete transforming growth factor beta 1, a powerful neuromodulator which rapidly suppresses spinal synaptic plasticity, DRG neuronal hyper-excitability, and neuropathic pain resulting from neuropathic injury. BMSCs that migrate to injured DRGs have been found to survive for 2 months, providing effective, and sustained analgesia.

The mechanisms of the analgesic effects conferred by BMSCs are mainly due to the paracrine factors secreted by the cells (Figure 1). BMSCs produce a large amount of biologically active molecules which regulate different functions through the interaction of different cell types (66). Herein, we will describe some of the major factors secreted by BMSCs that associated with analgesic actions.

### Transforming Growth Factor Beta

Transforming growth factor beta is a widely expressed secreted protein in various tissues that controls many cellular functions, including growth, proliferation, differentiation, and apoptosis. BMSC production of TGF-β has been shown by several investigators and immune modulation of BMSCs has been demonstrated to be partially mediated by TGF-β (14, 48, 61, 67). TGF-β is not only a powerful immunosuppressive cytokine but also a powerful neuromodulator. Previous studies have shown that BMSC administration can improve neurological function of animals with ischemic brain injury, with specific silencing of TGF-β abrogating the effects of the administered MSCs (68).

How does TGF-β1 reduce chronic pain? Accumulating evidence suggests that TGF-β1 inhibits nerve injury-induced activation and proliferation of microglia and astrocytes, and TGF-β1 also reduces the expression and secretion of pro-inflammatory cytokines (69–71). In our recent paper (14), we found that TGF-β1 can rapidly (within 1 min) modulate synaptic transmission in the spinal cord and neuronal excitability in DRG *via* a non-genomic mechanism. However, the mechanisms mediating TGF-β1 inhibition of neuropathic pain need to be further clarified. There is still controversy about the effects of TGF-β1 on chronic pain (72, 73). The results of different animal pain models are not consistent. In addition, TGF-β1 shows different activities on different cell types and even a single cell type at different stages of development. Identifying the details of the TGF-β1 analgesic signaling pathway will be important in developing our understanding of how TGF-β1 expression leads to analgesia in chronic pain conditions.

### Interleukin-10

Interleukin-10 is a powerful anti-inflammatory cytokine with multiple effects in immunoregulation and inflammation. The role of IL-10 in reducing chronic pain has been recognized and the secretion of IL-10 by BMSCs was investigated in several studies (48, 74, 75). Compared with high production of TGF-β, IL-10 release from BMSCs was very low (14). The role of IL-10 in BMSC-mediated analgesia remains controversial. Some authors have reported no significant changes in the level of IL-10 when using BMSCs and BMSC-conditioned media in different models *ex vivo* and *in vitro*. We found that IL-10 release from BMSCs did not contribute to BMSC-induced pain relief. The analgesic effect of BMSCs was neutralized by TGF-β1 antibody, but not IL-10 antibody (14). However, a recent study showed that the analgesic effects of intrathecally injected BMSCs were reversed by both TGF-β1 and IL-10 antibody neutralization (48).

### Tumor Necrosis Factor-Stimulated Gene-6 (TSG-6)

Tumor necrosis factor-stimulated gene-6 is a glycoprotein that was shown to produce potent anti-inflammatory effects. The relationship between BMSCs and TSG-6 has been intensively investigated (76). Therapeutic effects of BMSCs in some animal models of disease, such as cerebral ischemia (77), diabetes type 1 (78), peritoneal adhesions (79, 80), and experimental autoimmune encephalomyelitis (EAE) (81), was observed to be dependent on TSG-6. Cell tracking studies demonstrated that intravenously administered BMSCs mostly were trapped in the lung. Microarray analysis of BMSCs found in the lung revealed that one of the highest up-regulated transcripts was TSG-6. The silencing TSG-6 in BMSCs prior to administration resulted in loss of their therapeutic properties, while exogenous TSG-6 administration actually replicated the therapeutic activity provided by BMSCs (79).

### Hepatocyte Growth Factor 1 (HGF-1)

Hepatocyte growth factor 1 is a paracrine cellular growth, motility, and morphogenic factor, with a number of regenerative processes linked to its activation (82). Like other soluble factors that are implicated in regenerative processes, HGF-1 demonstrates immune modulatory activity. *In vivo* administration of HGF-1 has been shown to provide a protective effect from autoimmune disease, *via* activating regulatory T cells which produce the immunosuppressive cytokine IL-10 (83, 84). One of the key mediators responsible for the therapeutic activities of BMSCs *in vivo* is the production of HGF-1. When antibodies blocking HGF-1 are introduced, they cancel the protective effects of BMSCs and BMSC-conditioned media in the EAE model of multiple sclerosis (85). HGF-1 also appears to be necessary for the neuroprotective effects of MSC-conditioned media, demonstrated by experiments where apoptotic processes were not protected against in a glutamate-induced excitotoxicity model when HGF-1 function was neutralized (86). The HGF-1 generated from MSCs therefore shows a number of different effects, including angiogenesis, immune modulation, and protection from apoptosis.

### Metalloproteinases

Recently, human umbilical cord plasma was found to be enriched with tissue inhibitor of metalloproteinases 2 (TIMP-2) in a study that showed how systemic treatments of umbilical cord plasma and TIMP-2 increased synaptic plasticity and hippocampal-dependent cognition in aged mice (87). MMPs have been shown to play major roles in neuroinflammation and pain. MMP-9 produced by injured DRG neurons leads to the development of early-phase neuropathic pain through the activation of microglia, IL-1β cleavage, and microglial p38 activation. MMP-2 production leads to late-phase neuropathic pain caused by IL-1β cleavage and astrocytic ERK activation. TIMP proteins suppress neuropathic pain, with TIMP-1 alleviating early-phase neuropathic pain and TIMP-2 attenuating established late-phase neuropathic pain (88). The presence of TIMP-2 in umbilical cord plasma suggests the possibility that similar adult mesenchymal stem cell populations such as BMSCs may produce TIMP proteins which can likewise inhibit MMP-mediated neuroinflammation and pain.

## BMSCs in Clinical Practice for Pain Management

Transplantation of BMSCs is considered safe and has been extensively tested in clinical trials, including cardiovascular, neurological, and immunological disease, with exciting results. The growing interest in MSC therapy stems from their safety in treatments as well as the pleiotropic functions of MSCs that enhance endogenous repair mechanisms and attenuate immunological dysfunction. Currently, there are over 200 registered clinical trials sites worldwide for the evaluation of MSC treatment [http://clinicaltrials.gov/, summarized in Ref. (38)]. Investigators have also recognized the potential for BMSCs to treat painful diseases in patients (Table 1).

A pilot study (34) had 10 patients with degenerative disk disease and lower back pain receive autologous BMSCs. These patients reported a decrease in pain and disability at a level that was comparable with patients who received spinal fusion or total disk replacement surgery. Additionally, BMSC therapy offered the advantages of being a less invasive procedure and that helped to preserve the biomechanical functions of the lumbar spine. A study from Japan showed that degenerated intervertebral disks that received injections of autologous MSCs led to disk regeneration as well as reported alleviation of back and leg pain (89). In 2008, Centeno et al. discovered that the meniscus cartilage in a knee joint of an OA patient showed regeneration after intra-articular implantation of autologous MSCs (90). Subsequent safety reports for the use of MSCs in treating OA by Centeno’s group showed that follow-up MRIs in 227 patients at various time points ranging from 3 months to 2 years following implantation of BMSCs revealed no new tumor formations at the re-implantation sites (91).

To provide salvage treatment for cancer patients who received overdoses of radiotherapy, intravenous administration of allogeneic BMSCs from relatives of patients was performed in three patients with refractory and fistulizing colitis resembling fistulizing Crohn’s disease (37) and achieved a therapeutic effect. Systemic BMSC therapy of refractory irradiation-induced colitis is considered a safe and effective treatment for relieving symptoms, such as pain, inflammation, diarrhea, and hemorrhage. BMSCs also regulate immune response through an increase of T regulatory cells and a decrease of activated effector T cells (92). Encouraging results have been observed from clinical trials of Crohn’s disease, systemic lupus erythematosus, and systemic sclerosis (37, 38, 93). These data indicate that BMSCs offer a promising therapy strategy for a variety of immune-related diseases.

## Perspective

To date, the possible mechanisms of the analgesic effects mediated by BMSCs and their associated paracrine factors are still not very clear. Different types of growth factors, anti-apoptotic factors, anti-inflammatory and pro-inflammatory factors, and chemoattractants cause these effects. As more studies focus on each specific cytokine released by BMSCs, it has been demonstrated that blocking at least one of them leads to a decrease in the therapeutic effect. BMSCs seem to produce a complex network of cytokines, which can be found in BMSC-CM. This is why the effect of BMSC-CM is often similar to the effect of BMSCs themselves. BMSCs and BMSC-CM have clearly demonstrated measurable therapeutic effects in different models of acute renal, lung, hepatic, ischemia–reperfusion, and burn injury. It is important to continue these studies for further implementation of this therapy in clinical practice.

Recently, the paracrine functions of BMSCs have been found to be mediated, at least in part, by extracellular vesicles (EVs) (94, 95). EVs are mainly secreted from the endosomal compartment and contain contents from their cells of origin, such as miRNA, mRNA, and proteins (96, 97). Recently, animal model-based studies have shown that EVs have important potential as a novel advancement over whole cell therapies (98, 99). It is also important and necessary to understand the contents of EVs, the mechanism of EV exocytosis, and the therapeutic effects of EVs.

Unexpectedly, there are gender-dependent differences in the secretion of different cytokines by BMSCs *in vivo*. Crisostomo et al. (100) found that in BMSCs from female mice stimulated with LPS or hypoxia, the secretion level of VEGF was higher and the levels of TNF and IL-6 were significantly lower than in BMSCs from male mice. Additionally, in female mice, pro-inflammatory activity was lower while proliferative and regenerative activity was higher compared with male mice. Perhaps estrogen plays a role in these differences. It has been demonstrated that exogenous estrogen can increase the activity of MSCs and that MSCs express an estrogen receptor alpha, which may have played an important role in the proliferation and differentiation of MSCs (101). Further studies will be important to check for sex-dependent analgesic effects and signaling of BMSCs in chronic pain treatment.

## Conclusion

An increasing number of articles are being published that describe the anti-inflammatory effects attributed to BMSCs and their paracrine factors. With the association of neuroinflammation and various painful insults and pathologies, these studies demonstrate the ability of BMSCs to treat chronic pain. Due to their strong immunoregulatory properties and high expansion potential, BMSCs can be used for successful autologous and even heterologous transplantation. Therefore, injections of BMSCs may provide efficient, long-term, and safe therapy for patients with painful diseases.

## Author Contributions

All authors listed have made a substantial, direct, and intellectual contribution to the work and approved it for publication.

## Conflict of Interest Statement

The authors declare that the research was conducted in the absence of any commercial or financial relationships that could be construed as a potential conflict of interest.
